# The Impact of Non-Enzymatic Reactions and Enzyme Promiscuity on Cellular Metabolism during (Oxidative) Stress Conditions

**DOI:** 10.3390/biom5032101

**Published:** 2015-09-10

**Authors:** Gabriel Piedrafita, Markus A Keller, Markus Ralser

**Affiliations:** 1 Department of Biochemistry, University of Cambridge, 80 Tennis Court Rd, Cambridge CB2 1GA, UK; E-Mails: gp394@cam.ac.uk (G.P.); mk747@cam.ac.uk (M.A.K.); 2 The Francis Crick Institute, Mill Hill Laboratory, The Ridgeway, London NW1 7AA, UK

**Keywords:** oxidative stress, reactive oxygen species, metabolic damage, metabolite repair, enzyme promiscuity, underground metabolism

## Abstract

Cellular metabolism assembles in a structurally highly conserved, but functionally dynamic system, known as the metabolic network. This network involves highly active, enzyme-catalyzed metabolic pathways that provide the building blocks for cell growth. In parallel, however, chemical reactivity of metabolites and unspecific enzyme function give rise to a number of side products that are not part of canonical metabolic pathways. It is increasingly acknowledged that these molecules are important for the evolution of metabolism, affect metabolic efficiency, and that they play a potential role in human disease—age-related disorders and cancer in particular. In this review we discuss the impact of oxidative and other cellular stressors on the formation of metabolic side products, which originate as a consequence of: (i) chemical reactivity or modification of regular metabolites; (ii) through modifications in substrate specificity of damaged enzymes; and (iii) through altered metabolic flux that protects cells in stress conditions. In particular, oxidative and heat stress conditions are causative of metabolite and enzymatic damage and thus promote the non-canonical metabolic activity of the cells through an increased repertoire of side products. On the basis of selected examples, we discuss the consequences of non-canonical metabolic reactivity on evolution, function and repair of the metabolic network.

## 1. Introduction

During its lifespan, a cell is exposed to many hazards and changes in the environment. These include varying pH and temperature, osmotic imbalances, radiation, drought, starvation, contact with deleterious or toxic chemicals, or biotic interactions with other species. These stress situations can manifest as an acute pulse, or be sustained over time, and can affect cellular functioning and ultimately impair cell growth, replicative capabilities, and/or promote aging. Stress-induced damage to biological macromolecules such as DNA, proteins and membrane components has been intensively studied and characterized [1,2,3,4]. However, also cellular metabolites and the flux through metabolic pathways are affected. The impact of stress on metabolism and its small-molecule cellular constituents has attracted notable attention in areas of chemistry and toxicology [5,6], but only recently research has begun to systemically analyze the wide-ranging consequences at the systems level [7,8]. Since metabolism is a basal property of life and a main driving force during evolution, being the closest-to-phenotype cellular attribute, and given the reactive nature of many metabolites, the identification of stress-related metabolic processes appears to be of fundamental importance.

## 2. Chemical Damage to Metabolites: Oxidants and Antioxidants

**Figure 1:**
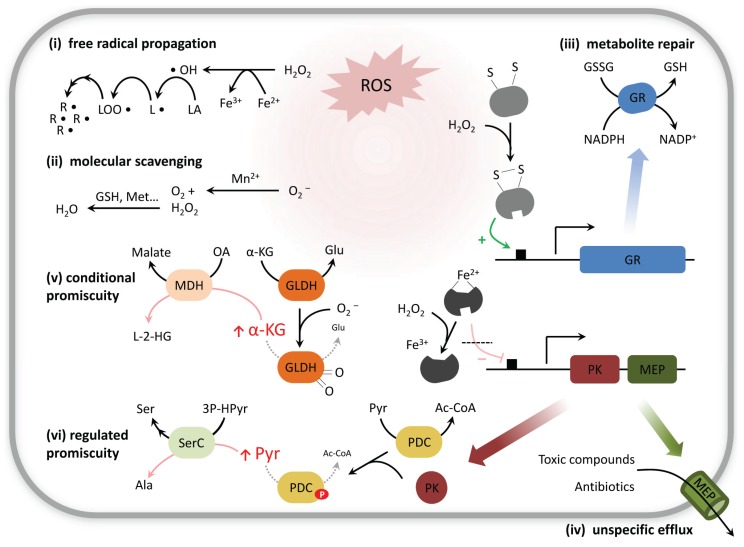
Formation of non-canonical metabolites and repair strategies. Chemical modification of metabolites leads to unwanted reactions, causing damage to both macromolecules and small molecules (**i**). Molecular scavengers cleanse and channel ROS toward less toxic products (**ii**). Stress sensors activate genes involved in specific protein-based repair responses (**iii**); or in unspecific transport processes (**iv**). As a general outcome, the metabolome reconfigures, affecting metabolite levels and fluxes; this in turn affects the specificity of enzymatic reactions. (**v**–**vi**) Selection of relevant examples from metabolism. Abbreviations: ROS, reactive oxygen species; Met, Methionine; GR, glutathione reductase; GSSG, oxidized glutathione or glutathione disulfide; GSH, reduced glutathione; MEP, multidrug efflux pump; LA, lipid; L•, lipid radical; LOO•, lipid peroxyl radical; R• complex radicals; GLDH, glutamate dehydrogenase; α-KG, α-ketoglutarate; Glu, glutamate; MDH, malate dehydrogenase; OA, oxaloacetate; L-2-HG, L-2-hydroxyglutarate; PK, protein kinase; PDC, pyruvate dehydrogenase complex; Pyr, pyruvate; Ac-CoA, acetyl-coenzyme A; SerC, phosphoserine transaminase; 3P-HPyr, 3-phosphohydroxypyruvate; Ser, serine; Ala, alanine.

One of the most important causes of metabolic damage is unspecific oxidation. Basal levels of oxidizing molecules arise constantly from normal cellular processes involving redox reactions or in the mitochondrial or photosynthetic electron transport chains [9]. Normal levels of oxidants are compensated by cellular metabolism, and being integral part of physiology, are required for biochemical reactions and as signaling molecules [10]. In a range of conditions referred to as “oxidative stress” [11], normal redox homeostasis is however disrupted and the balance between reducing and oxidizing molecules shifts towards the latter. Under oxidative stress, reactive oxygen species (ROS) represent one of the most important groups of cellular oxidants and comprise oxygen-derived molecules such as hydrogen peroxide (H_2_O_2_), superoxide anion (O_2_^−^), or hydroxyl radical (•HO). Non-enzymatic chemical reactivity plays a crucial role in this scenario, as for example in the context of the Fe(II)-catalyzed Fenton reaction, that converts H_2_O_2_ into •HO [12]. Superoxide anion and hydroxyl radical are highly reactive and indiscriminately damage metabolites, lipids, enzymes and nucleic acids via peroxidation [13,14,15]. This damage causes “errors” in metabolism [16,17], (Figure 1 (i)) such as the propagation of lipid peroxidation and carbohydrate oxidation [18,19]. In addition to the strong potential of superoxide anion to react with transition metals, it also combines with other chemical species, e.g., nitric oxide, and generates highly oxidant species such as peroxynitrite, causing further damage to lipids, altered nucleobases, and nitration of amino acid residues [20,21,22,23]. Under oxidative stress, the functionality of cellular systems declines, increasing the risk of cellular damage, apoptosis, aging, neurodegeneration and cancer [24,25,26].

Common targets to oxidative damage are amino acids, both in their free and protein integrated forms that are both highly abundant. Especially cysteine (which will be discussed later in the text) and methionine are the most prone to oxidation [27]. When the sulphur of methionine reacts with oxidizing molecules, it can form methionine-*S*- or methionine-*R*-sulfoxides, whose accumulation has been associated with various diseases and aging [28,29].

Biological systems exploit this property of metabolites and use them as scavengers for ROS. This applies not only to methionine and cysteine, but also to pyruvate, α-tocopherol, ascorbic acid, or other amino acids [12,30,31,32] (Figure 1 (ii)). While free methionine acts as general redox buffer, protein-coded methionine residues absorb oxidizing agents by positioning on the exposed protein surface and surrounding the active site, thereby preventing more severe oxidative damage on targeted proteins [33,34]. This oxidation of methionine residues at the protein surface can proceed without significant reduction in enzyme activity, as for example in glutamine synthetase, which is shown extremely resistant to H_2_O_2_ [33].

### 2.1. Not only Protein and DNA Damage, but also Metabolites Can Be Repaired

In analogy to DNA and protein repair, cells have developed a series of control mechanisms to cope with the oxidized/chemically damaged metabolites, summarized by the term “metabolite repair” [35] or “metabolite-damage control” [36,37,38]. These involve complementary strategies that either: (i) “repair” the chemical damage to metabolites (e.g., reversion through reduction of an oxidized molecule); (ii) degrade or convert the altered molecules into less harmful products or functional metabolites; or (iii) export the non-canonical metabolite. Some of these mechanisms are of general nature and overlap with the parallel occurring protein repair mechanisms (e.g., glutathione reductase, thioredoxin). Others are specifically targeted against small-molecule derivatives, as for example Glutathione peroxidase 4 (GPx4), which is a protein of the glutathione peroxidase (GPx) family with wide substrate specificity, able to reduce a variety of lipid hydroperoxides, preventing radical propagation through lipid peroxidation [39,40]. GPx4 activity obtains its redox potential through the reduced form of the tripeptide glutathione (GSH) [41,42], a molecular scavenger which is regenerated in a NADPH-dependent reaction catalyzed by glutathione reductase (GR) (Figure 1 (iii)). Indeed, most of the repair mechanisms require, directly or indirectly, energy supply in the form of ATP or reducing equivalents [4,15], and many are conditionally activated under stress conditions (Figure 1). The consequence is a sudden change of ATP and NADPH consumption once cells enter a stress situation and its balance requires metabolism to reconfigure.

**Figure 2:**
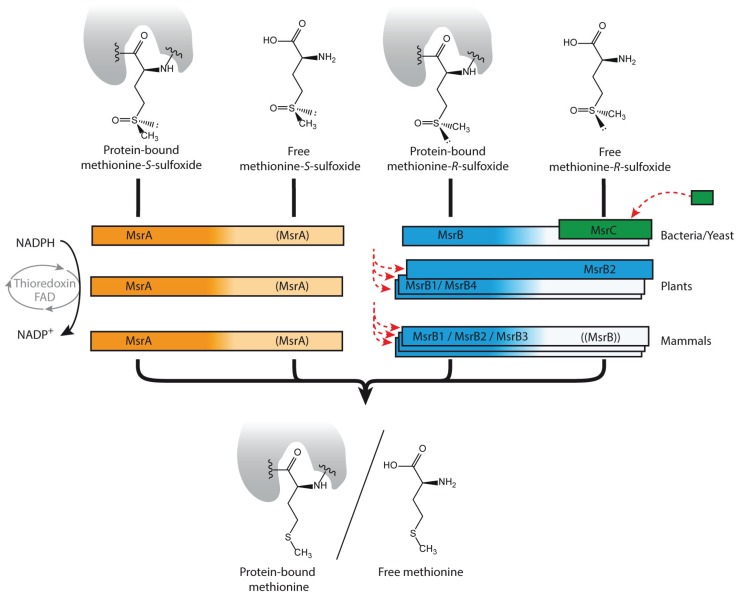
Oxidative damage on methionine and its repair enzymes. Methionine is oxidized to methionine-*S*-sulfoxide and methionine-*R*-sulfoxide derivatives. Three families of methionine sulfoxide reductases (Msr, different colors), revert the process depending on thioredoxin, each being stereo-specific against the *R*- or the *S*-enantiomeric form. While MsrA and MsrB are highly conserved with preference for protein integrated methionine sulfoxides, MsrC (f*R*Msr) is limited to unicellular organisms and reduces free methionine-*R*-sulfoxide. MsrB has evolved by duplication and divergence (red dashed arrows), accounting for coverage of diverse subcellular localizations, and enabling in plants the appearance of a subtype active against the free methionine-*R*-sulfoxide. Color intensities illustrate different strength in catalytic efficiency.

An illustrative example for specific oxidative stress-related repair concerns methionine. Repair of oxidized methionine does occur when the amino acid is in its free form as well as when it is part of a protein [43,44] (Figure 2). Three enzyme families of methionine sulfoxide reductases, MsrA, MsrB and MsrC, metabolize the derivatives in a stereospecific manner. Of them, MsrA and MsrB are highly conserved and reduce the *S*-sulfoxide and the *R*-sulfoxide residues, respectively, predominantly within a protein context [45]. MsrC (also named f*R*Msr) is only present in prokaryotes [46] and in some eukaryotes [47], and has its main affinity for free methionine-*R*-sulfoxide. Interestingly, MsrC exhibits no sequence homology with MsrA and MsrB, indicating independent evolutionary origin of these functionally overlapping proteins. In higher plants, where no specific MsrC-like activity exists, the repair function on free methionine-*R*-sulfoxide is efficiently covered by a subtype of MsrB proteins (which would have evolved by segmental duplication) [48], evidencing the evolution of alternative adaptive strategies to cope with a similar type of damage (Figure 2). As a common observation, repair mechanisms appear widely distributed, irrespective of whether metabolic damage proceeds through direct metabolite oxidation, non-enzymatic reactivity, enzyme inactivation, or enzyme promiscuity. Although most of these repair functions are non-essential under normal conditions, the respective mutants commonly show growth defects, lower survival rates or higher mutation frequencies [36,37,47,49,50]. In humans, deficiencies in metabolite detoxification systems are related to encephalopathic disorders [51], cancer predisposition [52], drug intolerance [53], or aging and a decreased lifespan [54]. Evidence has also been presented that neuronal clearance of toxic metabolites could be the explanation of why metazoans require sleep [55].

### 2.2. Other Forms of Metabolite Damage: Examples of Nucleotide and Cofactor Metabolism

Metabolism comprises a large diversity of molecules. The spectrum of possible combinatorial reactions and modifications on metabolites extends to a huge space of chemical reactions. Some modifications occur however more frequent than others. For instance, nucleotide metabolism is by its nature composed of many metabolites possessing high structural similarities. A range of abnormal nucleotides is generated as a consequence of spontaneous oxidation [56]. Others arise from unspecific enzymatic activities, as for example by methylation of nucleobases. Several of these aberrant compounds with only slight structural differences from the standard bases, can subsequently be recognized by downstream enzymes which propagate the error via incorporation. The mischarge of nucleoside diphosphate (NDP) kinases, which can promiscuously accept a broad range of base-differing substrates, releases abnormal NTPs that cause mispairing and mutagenesis if incorporated into DNA [56,57]. Cells have developed various families of NTP pyrophosphatases that intercept and hydrolyze non-canonical NTPs [37]. For instance, MutT-like proteins of the widespread Nudix superfamily catalyze—with variable degree of substrate specificity—the detoxification of oxo-purine dNTPs generated during oxidative stress, and thereby prevent DNA damage [58,59,60]. In other cases, substrate promiscuity (which will be discussed in more detail below) is the main contributor to the pool of abnormal nucleotides: dUTPases hydrolyze with high specificity dUTP, discriminating against normal cytosine and thymine nucleobases. dUTP can arise from UDP through a secondary activity of ribonucleotide reductase followed by phosphorylation, or by (catalytic) deamination of a deoxycytidine (tri-)phosphate. Interestingly, this principle of dUTPase activity is present in all three kingdoms of life, but is fulfilled by two mutually exclusive dUTPase families that present no sequence homology, yet being functionally analogous [61,62].

Another quantitatively highly relevant case concerns chemical modification of enzymatic coenzymes, such as NADH or FADH. Hydration of NADH or NADPH is common, occurring either non-enzymatically [63] or as a side reaction of promiscuous enzymes such as glyceraldehyde 3-phosphate dehydrogenase [64]. The resulting *R* and *S* hydrated forms of NAD(P)H display high structural similarity with the cofactors but are non-functional and inhibit various dehydrogenase reactions [65]. An ADP/ATP-dependent NAD(P)H dehydratase, widespread in all domains of life, converts the *S* enantiomeric derivative back to NAD(P)H [63]. Its action is complemented by the catalytic activity of an epimerase that catalyzes the interconversion between the *R* and *S* hydrated forms, an enzyme that appears fused to the dehydratase in some species such as *Escherichia coli*. Interestingly, in higher plants [66,67] and also in mammals [68] both genes possess organellar targeting sequences, allowing the proteins to be directed and co-localize within diverse subcellular compartments.

## 3. Non-Enzymatic Metabolic Reactions Are Affected by Stress Conditions

A second aspect of the susceptibility of metabolism concerns non-enzymatic reactions that occur as part of normal metabolism or through non-specific chemical reactivity but that contribute to canonical metabolic pathways [69,70]. Such non-enzymatic reactions are of high importance for the evolution of metabolism, as exemplified by the metabolism-like non-enzymatic interconversions that can occur between central carbon metabolites, which might have given rise to the early evolution of glycolysis and pentose phosphate pathway [71]. The presence of an enzyme catalyst in modern cells does however not prevent these non-enzymatic chemical reactions to occur in parallel to the enzymatic pathways. Termed Class I non-enzymatic metabolic reactions, a subset of non-enzymatic reactions are of low substrate specificity and can affect multitude of metabolites and modify a series of macromolecules [69]. These include Maillard reactions, unspecific conjugations of amino acids that are accelerated under heat and UV exposure, and also unspecific protein modifications such as protein (poly-)phosphorylation, glycosylation or acylation. The term “underground metabolism” [70] has been suggested to summarize the repertoire of this chemical reactivity, both non-enzymatic and enzyme-promiscuous, occurring in parallel to the flux of functional metabolic pathways. This reactivity is distinguishable from other more specific non-enzymatic reactions that are part of the metabolic network, and occur either exclusively non-enzymatically (Class II non-enzymatic metabolic reactions) or in parallel to existing enzyme function (Class III) [69].

Non-enzymatic reactions are dependent on the chemical environment (metal availability, pH, temperature, ionic strength), and must therefore be particularly sensitive to stress conditions. This is well illustrated for the case of non-enzymatic protein acylation: A number of essential, endogenous thioester metabolites, such as acetyl-CoA or succinyl-CoA, can unspecifically cross-acylate protein lysines [72,73]. Protein acylation appears most prominent in mitochondria, correlates with the levels of these reactive metabolites and with mitochondrial energy-metabolism, and has been linked to a form of “carbon stress” [74]. Non-enzymatic modification of macromolecules can alter protein function and may require quality-control responses. In this case, the sirtuin family of deacetylase enzymes (which has received notable attention by a potential connection to the benefits arising from a calorie-restriction diet) has been proposed to counteract the potential deleterious effects of non-enzymatic protein acylation and thus to play a role in stress resistance [74]. Similarly, for small molecules, intermediates generated in non-enzymatic reactions can, if accumulated, interfere with metabolic pathways by acting as competitive inhibitors of enzymes, or serve as alternative substrates, and thus need to be cleared.

While specific repair mechanisms might have evolved for non-canonical metabolites which are produced in higher quantities or present strong cytotoxic effects, it is reasonable to assume that not all metabolic side products possess specific clearance mechanisms. This becomes illustrative, as the number of potential chemical reaction products from non-enzymatic reactivity exceeds the number of enzymes encoded in a typical genome by several orders of magnitude. A broad range of metabolites, including especially those for which no specific cleaning enzyme exists [75,76,77], might however be cleared via unspecific cellular export, which is largely mediated by efflux pumps and transmembrane channels such as multidrug transporters (Figure 1 (iv)). In bacteria, numerous studies report the involvement of membrane transporters in multidrug resistance [78,79,80]. *E. coli*’s resistance to a high number of compounds is mediated by the outer-membrane pore-forming protein TolC [81]. This transporter acts in concert with the inner membrane TolC-dependent efflux pump AcrB and with cognate periplasmic proteins (e.g., AcrA) to form tripartite trans-periplasmic exporters that push xenobiotics out of the cell [82,83]. There is growing evidence suggesting that TolC-mediated extrusion is not limited to xenobiotics [84]: *E. coli* tolC mutants show lower fitness phenotypes in certain stress conditions [85], accumulate cell-synthesized enterotoxins and potentially-deleterious intermediary metabolites [86], and overexpress key stress response regulators, presumably triggered by the abnormal retention of toxic cellular products [87]. Similar transport mechanisms operate in many other bacteria [80].

## 4. Enzymes That Change Substrate Specificity during Stress Conditions

**Figure 3:**
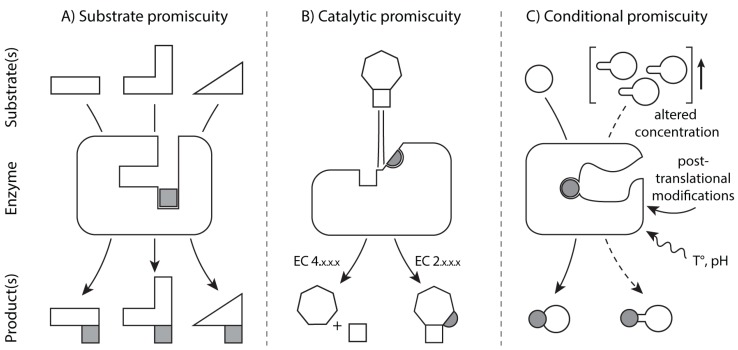
Mechanistic classification of enzyme promiscuity. (**A**) Substrate promiscuity or multispecificity: A certain enzyme can perform the same catalytic reaction on a diverse set of substrates indistinctly; (**B**) Catalytic promiscuity: Different chemical transformations are allowed by the same enzyme, according to which this can be classified with various E.C. numbers. (**C**) Conditional promiscuity: Latent secondary activities of an enzyme might gain activity in response to environmental changes, e.g., due to an increase in the concentration of substrate analogs with lower affinity for the enzyme, or by post-translational signals related with induced conformational changes. The three examples shown are merely illustrative but inspired by transaminase TyrB, cytosine methyltransferase and thymidine kinase, respectively.

A third important aspect of metabolism during stress conditions does not concern the direct chemical modification of metabolites, but affects them indirectly by altering enzyme function. Upon damage, several enzymes decrease in substrate specificity and simultaneously increase in promiscuity. When enzymes are modified, novel subsidiary activities and interactions can arise concomitantly from the altered proteins, enabling additional metabolic reactions, some of potential physiological significance [70,88]. It is a general property of metabolism that most metabolic enzymes are not as specific as sometimes depicted in textbooks, and are in fact error-prone or ambiguous in their function [89,90] (Figure 3) (The concept of “enzyme promiscuity” has been massively used recently, but in different contexts and with different meanings [91]. The classification that we make below follows that by Hult and Berglund [91].).

Most enzymes accept multiple substrates and possess affinities for a wider range of compounds, a phenomenon referred to as substrate promiscuity (Figure 3A) (Some authors consider that the capacity of an enzyme to catalyze various natural substrates may be more conveniently described by the term “substrate multispecificity” or “cross-reactivity”, so that the term “substrate promiscuity” is reserved to adventitious secondary activities different from the one the enzyme has evolved: see [90,92].). A recent example of notable attention is the enzyme TP53-induced glycolysis and apoptosis regulator (TIGAR), an enzyme that possesses an important role in cancer cell metabolism. TIGAR was first identified as fructose-2,6-bisphosphatase (F26BPase) [93], but it accepts multiple substrates. TIGAR shows its highest activity as phosphoglycolate-independent 2,3-bisphosphoglycerate phosphatase [94], acting most efficiently on a metabolite whose function is so far unclear.

Other enzymes are able to catalyze more than one type of chemical reaction on a given set of substrates (catalytic promiscuity, Figure 3B). This is the case of certain cytosine methyltransferases that are able to catalyze cytosine methylation as well as cytosine deamination. Cytosine deamination yields thymine, implying that this catalytic activity causes mutations and could play a potential role in tumourogenesis [95].

A classical example of enzymes that possess promiscuous activities that can lead to toxic by-products are malate dehydrogenase (MDH) and isocitrate dehydrogenase (IDH1 and IDH2). MDH typically catalyzes the interconversion between malate and oxaloacetate, but possesses certain affinity towards α-ketoglutarate. Despite the much higher affinity for the canonical substrate, high intracellular levels of α-ketoglutarate promote that MDH also produces l-2-hydroxyglutarate (see conditional promiscuity, Figure 1 (v), Figure 3C) [35]. A widely distributed FAD-dependent enzyme, the l-2-hydroxyglutarate dehydrogenase, specifically converts this side-product back into α-ketoglutarate [36]. Deleterious mutations in this enzyme in humans are responsible for an inherited metabolic disease called l-2-hydroxyglutaric aciduria, attributed to the accumulation of the aberrant product [51]. An analogous detoxification system exists also for its enantiomer d-2-hydroxyglutarate [96]. This normal metabolic intermediate is overproduced by mutant forms of isocitrate dehydrogenase-1 and -2 (IDH1 and IDH2) [97]. These mutations, preferentially occurring in cancer, alter the enzyme towards an increased production of this metabolite, which is otherwise formed in a side reaction and at a low rate only. d-2-hydroxyglutarate is also found at increased levels in patients suffering from the metabolic syndrome d-2-hydroxyglutaric aciduria [98], a genetically heterogeneous neurometabolic disorder attributed to germline mutations in IDH2 [99] and mutant genotypes of the d-2-hydroxyglutarate dehydrogenase, the enzyme responsible for d-2-hydroxyglutarate repair [98].

Importantly, such secondary activities of enzymes are often latent in normal growth conditions, but can become relevant in response to environmental changes or stresses (conditional promiscuity, Figure 3C), either by: (i) an enhanced bioavailability of a low-affinity substrate analog in comparison with the concentration of the natural substrate; (ii) conformational (allosteric) changes of the enzyme induced by direct exposure to stressors; (iii) conformational changes regulated by specific post-translational modifications exerted by stress-related signaling pathways. Therefore, enzyme promiscuity must be of critical importance for an integral view of metabolic reconfiguration under stress conditions (Figure 1).

An illustrative case is represented by metalloenzymes that exhibit a tendency toward mis-metallation during stress conditions. The activity of most metalloproteins depends on the binding of the correct metal, with a mis-metallation resulting in change of reactivity, or partial to total inactivation. Bacterial ribulose 5-phosphate epimerase (Rpe) and some other enzymes that are canonically iron-dependent, actually accept both iron Fe(II) and manganese Mn(II) as cofactor [100,101]. As pointed out by Cotruvo and Stubbe [102], Fe(II)- and Mn(II)- binding affinities are comparable in these proteins, and thus the discrimination between both metals is roughly determined by the differential bioavailability. While the enzymes are hence bound to Fe(II) under normal cellular growth conditions, they appear charged with Mn(II) under oxidative stress or iron-limiting conditions. This ambiguity is regarded as an adaptive strategy to protect enzymes from irreversible inactivation and severe damage [100] (Table 1): (i) Fe(II) centers are sensitive to ROS and oxidize to Fe(III), causing enzyme inactivation [101]; (ii) free iron pools react with ROS propagating the toxic free radical formation [103]. Interestingly, the same transcriptional control mechanisms that are responsible to ensure iron sequestration and control during oxidative stress [103] are linked to Mn(II) import and the promotion of the Mn metabolism [102]. Noteworthy, Mn(II), when bound to phosphate or carbonate, can act as an efficient non-enzymatic catalyst for the superoxide disproportionation at physiological conditions [104], which can in fact rescue yeast superoxide dismutase-knockouts [105], conferring an additional role to Mn(II) as antioxidant.

### Adaptive Promiscuity: When Altered Substrate Specificity Becomes Advantageous

Despite being an energetic burden to the cell, promiscuity and multi-substrate specificity is an integral biological property in metabolic networks, and is essential as it is necessary for the evolution of metabolism [106]. In some instances, the ability to process multiple substrates is beneficial to cells in stress situations (Table 1). Perhaps one of the most illustrative examples is the genetic code “ambiguity”. Translational fidelity, and thus an adequate protein biosynthesis, relies on the accurate aminoacylation of the different tRNA molecules by specific aminoacyl-tRNA synthetases and on unambiguous codon-anticodon pairing within the ribosome. Yet a certain degree of permissiveness is known to exist due to nucleobase wobbling, accounting for the degeneracy in the genetic code. Indeed, numerous exceptions and variants to the standard decoding have been reported in different organisms [107,108,109]. Netzer *et al.* [110] demonstrated how the genetic code accuracy can also change within the lifespan of an individual cell, as a consequence of induced changes in the acylation fidelity of aminoacyl-tRNA synthetases. A small but significant amount of methionine (Met) residues (approximately 1% of all coded Met) misacylate to non-cognate tRNA in normal unstressed mammalian cells. This fraction sharply increases (to approximately 13%) under ROS-related stress conditions, for example when exposing cultured cells to viral infections, bacterial wall factors or physico-chemical stressors. This results in proteins with high content in mistranslated Met. As discussed above, Met is a biological scavenger of ROS, proposing an adaptive role of methionyl-tRNA synthetase (MetRS) promiscuity and controlled Met-misacylation in coping with oxidative damage (Table 1). A recent study [111] reveals that the modulation of MetRS specificity is mediated by particular serine phosphorylations driven by the protein kinase ERK, which is a common mediator in the signaling response to stress. This represents a further indication about the *in vivo* protective role of Met-misincorporation for cell survival and growth under ROS stress.

The antioxidant function of Met residues might be also the cause of AUA codon reassignment in mitochondria, from isoleucine (Ile) to Met, which is widespread in vertebrates and also occurs in several invertebrates with a highly aerobic metabolism (like insects) and in some unicellular eukaryotes, including *Saccharomyces cerevisiae* [112]. With the respiratory chain as continuous source of ROS, one might hypothesize that a permanent redefinition of the genetic code (with one extra codon assigned to Met, and 2 instead of 3 codons for Ile) could have been a more suitable evolutionary adaptation in mitochondria than an inducible mechanism to tune promiscuous methionylation activities on non-methionyl tRNAs. Integral proteins of the mitochondrial inner membrane are overloaded with Met on their exposed surfaces (a classical preferential position for Ile residues), and in this way a significant containment of oxidative damage is achieved [112].

**Table 1:** Possible physiological benefits of various promiscuous and redundant activities induced in response to stress or environmental changes.

Stress	Enzyme	Induced Activity	Alleged Function	Reference
Oxidative stress	Mammalian MetRS	Mismethionylation of non-cognate tRNAs	Protect enzymes from inactivation	[110]
Biotic interaction	SerRS/LeuRS from *Candida*	Misleucinylation of Ser-tRNA	Increased invasivity, immune invisibility	[113]
Biotic interaction, anaerobiosis, starvation...	Bacterial transaminases	Redundancy in Ala & Lys biosynthesis	Robust peptidoglycan biosynthesis	[114]
Oxidative stress	Bacterial Pentosephosphate epimerase (Rpe)	Mismetallation with Mn(II)	Protect enzymes from inactivation	[102]

Another case in which ambiguous tRNA acylation has been proven beneficial is that of the fungus *Candida albicans* [115] (Table 1). Here the codon CUG can be translated both as a leucine (Leu) and as a serine (Ser) (with 5% and 95% frequency, respectively). This duality arises from the capacity of two aminoacyl-tRNA synthetases (LeuRS and SerRS) to recognize the particular unique serine tRNA (CAG) and allows statistically distributed populations of different mistranslated proteins from the same gene. This increase in phenotypic diversity is exploited in pathogenicity [113]. In fact, genes encoding cell surface adhesins are especially rich in CUG codons: partial CUG mistranslation as Leu results in morphological variations and heterogeneity in the cell wall that modify *Candida* interactions with the host microenvironment. In particular, experimental evidence shows that adherence capacities improve while recognition by the hosts immune system decreases [113], both being related with *Candida* virulence.

## 5. Changing Activity and Metabolic Switches: Stress Situations That Affect Flux Distribution

Enzyme activities are tunable and the degree of affinity and/or efficiency (*K*_m_, *k*_cat_) for different metabolites may change according to conditions [91] (Figure 3C). This also applies during the individual lifetime of an enzyme, e.g., due to age related conformational alterations [116]. As a consequence, aside from the immediate effects of protein damage on the enzymes natural function and its promiscuous activities, the inactivation of a metabolic enzyme commonly involves an adaptation in total metabolic flux and/or results in a redistribution of flux toward other pathways. Due to its redox capacity, cysteine plays a pivotal role in many enzyme catalytic mechanisms, and the redox sensitivity of this amino acid renders several enzymes vulnerable to redox imbalances and ROS accumulation [27,117,118]. Interestingly, this redox sensitivity is exploited by cells to intensify metabolic protection in stress situations. In yeast, two enzymes of glycolysis, glyceraldehyde-3-phosphate dehydrogenase (GAPDH) and pyruvate kinase (PK), have been shown to be sensitive to oxidation, leading to a rapidly induced (in seconds-scale) but temporal glycolytic block [119,120]. Termed the glycolysis/PPP transition, the oxidative part of the pentose phosphate pathway (PPP) that is more redox resistant, increases in activity, resulting in enhanced NADPH production under the conditions where this cofactor is required by the anti-oxidant machinery [119,121]. The inactivation of GAPDH during oxidative stress is not only an adverse side reaction; the enzyme possesses evolved structural features that compete for free H_2_O_2_ to assure rapid inactivation when levels of this metabolite rise [122]). Increased PPP activity and stress resistance augmentation in yeast are also observed when the activities of triosephosphate isomerase [123] and pyruvate kinase [124,125] are reduced by genetic modification, implying that increased NADPH production in the oxidative PPP is a recurrent consequence to blocks in glycolysis. Recently, time-resolved metabolomics has revealed this mechanism to be conserved in mammalian skin cells, revealing however that the mechanistic trigger of the glycolytic/PPP transition is not yet understood [126]. In this context, it is however important to keep in mind that the contribution of distinct metabolic pathways, including the oxidative PPP, to the total NADPH pool differs between yeast and mammalian cell types, and even between tissues [127], so that the individual cells and organisms may distribute NADPH production in a different manner.

Another oxidant-induced change in metabolic flux distribution is connected to the polyamine metabolites spermine, spermidine and putrescine. These polycations are implicated in a multitude of cellular functions, including the stress response [128]. Treatment with peroxides affects the intracellular concentrations of spermidine and putrescine in correlation with the expression of the polyamine transporter *TPO1* [129]. Yeast cells overexpressing this transporter are sensitive to oxidants and are unable to induce canonical stress response proteins, such as HSP70 or HSP104. Effects of the transporter overexpression on metabolite levels are only detected upon application of the oxidant; stress-induced transport of these metabolites thus appears critical for the interaction of the metabolome and the proteome in stress situations [129]. In this respect, polyamine catabolism has attracted attention as a potential contributor to cytotoxicity, with H_2_O_2_ being a product of polyamine oxidation [130]. Studies in human breast cancer cell lines demonstrate the involvement of the polyamine degradative enzyme spermine oxidase SMO (PAOh1) in the production of H_2_O_2_ upon induction by a spermine analogue [131]. Overexpressing SMO leads to sub-lethal DNA damage and this confers sensitivity to radiation [132,133].

## 6. Evolution and the Impact of Metabolic Inaccuracy on the Genotype to Phenotype Relationship

Carbonell *et al.* [134] implies that promiscuous enzymes are widespread in the phylogenetic tree of life and appear statistically more shared across species (allegedly more ancient) than specific ones, consistent with the hypothesis of an evolutionary trend toward the specialization of enzymes [135,136]. The different levels of specificity of current metabolic enzymes would have evolved by segmental duplication of previously promiscuous protein-coding genes. Enzymes would diverge at different rates depending on trade-offs and selective pressures over their particular function and the environmental context [106]. Interestingly, the content in promiscuous and unspecific enzymes differs between organisms and correlates with their ecology; taxonomic groups facing stronger environmental changes possess more promiscuous enzymes [134]. Recently, evidence has been provided that a fraction of latent enzymatic side activities maps to functional products of the existing metabolic network, and confers advantage under novel growth environments [137].

**Figure 4:**
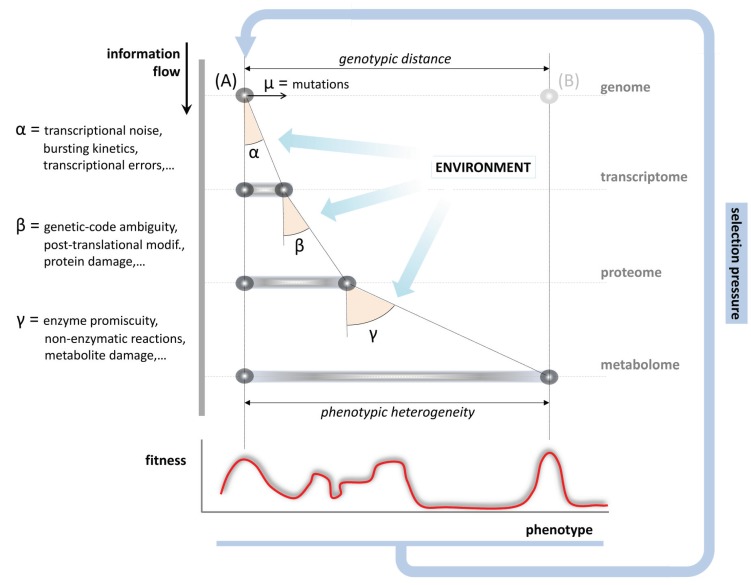
Impact of metabolic inaccuracy on the genotype-to-phenotype relationship. Biological processes, including transcription, translation and metabolism are not totally accurate. During the gene-protein-metabolite information flow, errors generated at each operational layer that are not compensated (depicted as angles α, β, γ) propagate, overall shaping the phenotypic space to be achieved from the same genotype (variation in the x-axis). Phenotypic variability arising due to error propagation can be enhanced by environmental stress conditions and enables a wide space of positions in the fitness landscape (red curve), all of which contribute to define the selection pressure over the given genotype (**A**). Promiscuity, noise, and unspecificity may thus facilitate the physiological adaptation to changing conditions, which would otherwise require the evolution to a different genotype (**B**).

Also metabolite repair—potentially in the form of much more rudimentary mechanisms—might date back to the very early stages of evolution and could already have been important for the development of metabolism during the origin of life [138], as first metabolic networks likely required mechanisms to achieve a minimally robust functioning [139]. During subsequent evolution, non-enzymatic reactions and enzyme promiscuity did contribute to the increase in the phenotypic variability of a cell. Persisting in modern cells, metabolic ambiguity is to be added to the sources of variation at the transcriptional and translational levels, required to understand the genotype-to-phenotype relationship (Figure 4): During the flow of information from gene over protein to metabolite, errors and variability are propagated, increasing the spectrum of phenotypes to be reached from the same genotype. Whereas most of these variants may involve a fitness cost for a well-adapted organism, flexibility facilitates adaptation when conditions change (Figure 4).

## 7. Conclusions

With the advent of systems biology and analytical techniques that allow analyzing multiple cellular metabolites in parallel, it has become clear that metabolism is constituted by a highly dynamic network. In this short review, we discuss examples that reveal the impact of stress conditions on metabolites, metabolic reactions and metabolic flux. Non-enzymatic reactions, chemical reactivity between metabolites, and enzyme promiscuity are inevitable consequence of the chemical properties of essential metabolites, and mechanistic and structural constraints of the participating enzymes. In stress conditions, these factors play synergetic roles in the propagation of errors and damage within metabolism, but also form the basis to reconfigure metabolism so that cells can adapt to changing or stress-inducing environments [90,140].

Metabolism in stress situations appears to be mainly affected by: (i) direct chemical modification of metabolites; or (ii) damage on enzymes that change their substrate specificity and/or catalytic activity. Temporary blockage of metabolic pathways translates as changes in the metabolic flux distribution. Although stress situations appear to be deleterious in the first instance, they have been exploited by evolution to extend the metabolic network, and to develop dynamic response mechanism to cope with changes in the environment. This includes either the use of promiscuous metabolites in signaling, so that repair and damage responses are initiated and function as stress sensors [10,27,141], or direct defense systems that involve stress-induced redistribution of the metabolic flux [70,119,120]. Finally, enzyme conditional promiscuity acts as a reservoir of new functions that can evolve into tunable mechanisms [142], with some of the non-canonical metabolites turning out to be useful [70,106,143,144,145]. Metabolism during stress conditions thus changes in activity, structure and complexity; and contributes to evolution, phenotype, and survival in stress situations.
